# Capturing the Fantastic Voyage of Monocytes Through Time and Space

**DOI:** 10.3389/fimmu.2019.00834

**Published:** 2019-04-16

**Authors:** Ye Chean Teh, Jeak Ling Ding, Lai Guan Ng, Shu Zhen Chong

**Affiliations:** ^1^Functional Immune Imaging, Singapore Immunology Network (SIgN), A^*^STAR (Agency for Science, Technology and Research), Biopolis, Singapore; ^2^Department of Biological Sciences, National University of Singapore (NUS), Singapore, Singapore; ^3^Department of Microbiology & Immunology, Immunology Programme, Life Science Institute, Yong Loo Lin School of Medicine, National University of Singapore, Singapore, Singapore; ^4^School of Biological Sciences, Nanyang Technological University, Singapore, Singapore

**Keywords:** monocytes, marginal pool, bone marrow, spleen, CXCR4 = chemokine receptor 4, inflammation, steady-state, intravital 2P microscopy

## Abstract

Monocytes are a subset of cells that are categorized together with dendritic cells (DCs) and macrophages in the mononuclear phagocyte system (MPS). Despite sharing several phenotypic and functional characteristics with MPS cells, monocytes are unique cells with the ability to function as both precursor and effector cells in their own right. Before the development of hematopoietic stem cells (HSCs) *in utero*, monocytes are derived from erythro-myeloid precursors (EMPs) in the fetal liver that are important for populating the majority of tissue resident macrophages. After birth, monocytes arise from bone marrow (BM)-derived HSCs and are released into the circulation upon their maturation, where they survey peripheral tissues and maintain endothelial integrity. Upon sensing of microbial breaches or inflammatory stimuli, monocytes migrate into tissues where their plasticity allows them to differentiate into cells that resemble macrophages or DCs according to the environmental niche. Alternatively, they may also migrate into tissues in the absence of inflammation and remain in an undifferentiated state where they perform homeostatic roles. As monocytes are typically on the move, the availability of intravital imaging approaches has provided further insights into their trafficking patterns in distinct tissue compartments. In this review, we outline the importance of understanding their functional behavior in the context of tissue compartments, and how these studies may contribute towards improved vaccine and future therapeutic strategies.

## Introduction

When agent Grant was traveling through the blood vessels of Dr Jan Benes in the science fiction movie “*Fantastic Voyage*,” he might have noticed a large white blood cell with abundant cytoplasm and a hefty eccentrically placed kidney bean-shaped nucleus. This cell measured approximately 20 μm in diameter and was the largest of all circulating leukocytes. Known as the monocyte, this cell is renowned for its phagocytic activity and constitutes about 5–10% of total blood leukocytes.

For half a century, monocytes were touted to be an intermediate cell type with the sole purpose of replenishing tissue macrophages (1, 2). This dogma was based on Van Furth and Cohen's findings in the mid twentieth century (3, 4) and has been a subject of intense research and debate in the past decade. While genetic fate-mapping experiments have since revealed embryonic progenitors as the precursors of most tissue macrophages (5–7), it is increasingly apparent that these original theories are not entirely incorrect either. Instead, it is now proposed that monocytes have the ability to reconstitute the macrophage pool, in a temporal and spatial manner (8, 9), with competition for a restricted number of niches as the main driving factor (10).

**Figure 1:**
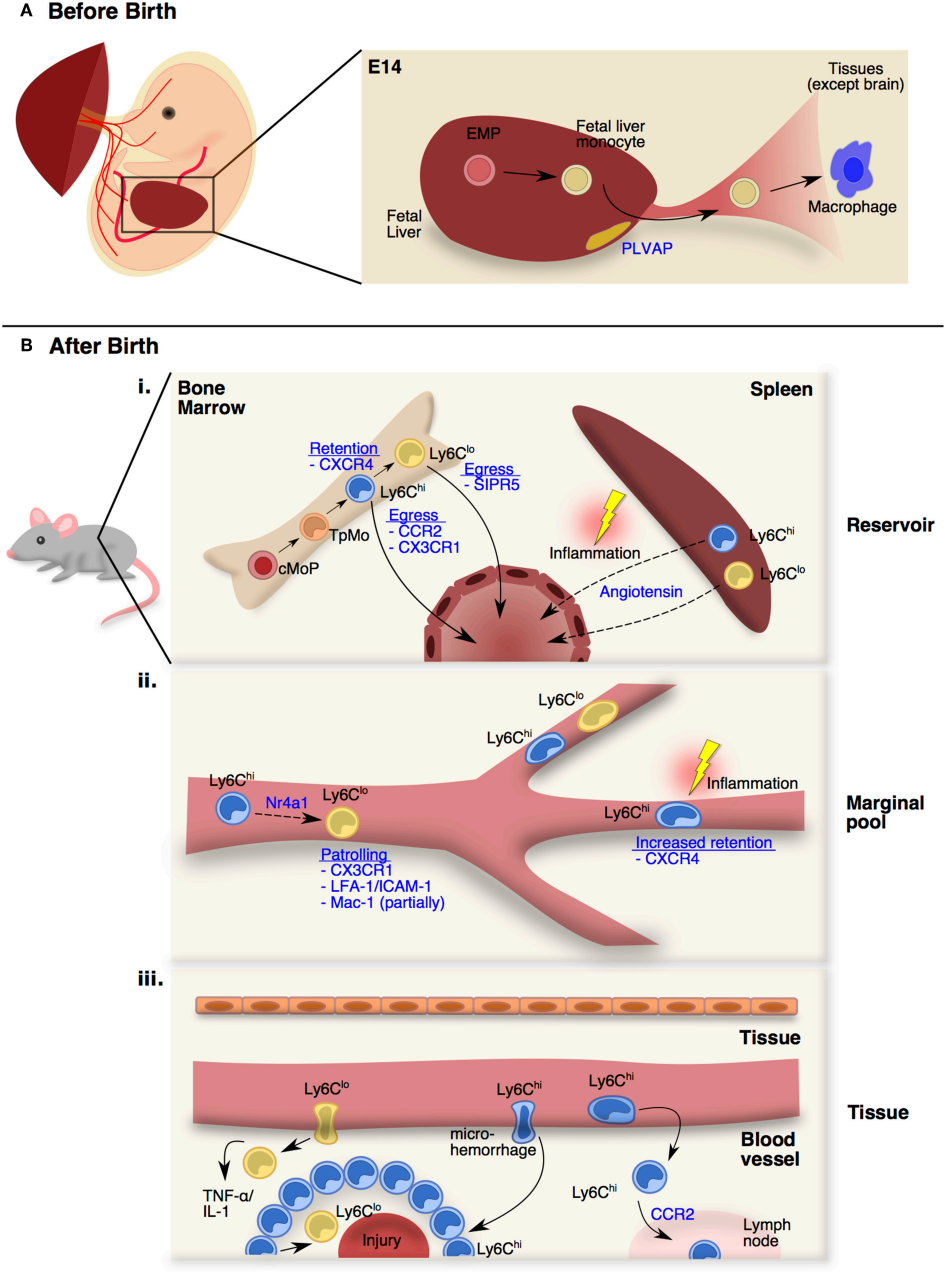
Monocyte trafficking and function in distinct stages and peripheral sites. **(A)** From E13.5 onwards, fetal monocytes derived from erythro-myeloid precursors (EMPs) in the fetal liver can be released into the circulation in a plasmalemma vesicle-associated protein (PLVAP) dependent manner. At E14.5, these fetal monocytes will colonize the open niches of every tissues as fetal monocyte-derived macrophages except the brain. **(B)** After birth (i) Adult monocytes originate from the common monocyte progenitors (cMoPs) that give rise to Ly6C^hi^ monocytes through a transitional precursor called transitional pre-monocytes (TpMos). Ly6C^hi^ monocytes are released into the circulation upon their last division, and differentiate into Ly6C^lo^ monocytes. The retention and egress of Ly6C^hi^ monocytes are dependent on CXCR4- and CCR2-signaling respectively, whereas Ly6C^lo^ monocytes egress is dependent on S1PR5-signaling. At steady state, circulating monocytes enter the spleen as a secondary reservoir. During inflammation, splenic Ly6C^hi^ and Ly6C^lo^ monocytes are mobilized into the circulation via Angiotensin-II/AGTR1A-signaling. (ii) Upon entering the circulation, short-lived Ly6C^hi^ monocytes gradually differentiate into longer-lived Ly6C^lo^ monocytes via Nr4a1-signaling. Ly6C^lo^ monocytes patrol the vessels partially via Mac-1, but significantly via CX3CR1-signaling and LFA-1/ICAM-1 interaction with the endothelial cells. At steady state, Ly6C^hi^ monocytes do not interact closely with the endothelium except in the vascular beds of distinct peripheral organs. CXCR4 regulates steady state monocyte margination in the lung. During inflammation, Ly6C^hi^ monocytes increased their transit time, resulting in increased retention in the microvasculature. (iii) At steady state, Ly6C^hi^ monocytes survey the tissue environment for antigens to transport into draining lymph nodes. During injury, Ly6C^lo^ monocytes infiltrate rapidly into inflamed site to provide TNF-α and IL-1. Besides the classical rolling and migration steps, a proportion of Ly6C^hi^ monocytes utilizes microhemorrhages to extravasate and enter inflammatory sites rapidly and form a ring-like structure before differentiating into Ly6C^lo^ monocytes for tissue repair.

With monocytes no longer functioning solely as steady-state macrophage precursors, it remains unclear what tasks they may perform in immunity and host defense. Monocytes are heterogeneous and consist of a classical population (Ly6C^hi^ in mice; CD14^++^CD16^−^ in humans) and a non-classical population (Ly6C^lo^ in mice; CD14^+^CD16^+^ in humans) (7, 11, 12) with distinct functional roles (13). Interestingly, amidst the flurry of excitement in examining macrophage ontogeny by genomics/epigenomics approaches, the understanding of monocyte function in the context of spatial distribution and tissue niche was also steadily emerging as a key focus area. Together with the development in molecular and cell biological studies (14), the advent of imaging techniques such as two-photon intravital microscopy (2P-IVM), which allows direct visualization of immune cells using fluorescent reporter-tagged mice *in vivo* and *in situ* (15, 16), has helped to uncover a wide array of imperative monocyte biology. Nevertheless, monocyte behavior is highly distinct in each tissue compartment due to their plasticity and sensitivity to niche signals (17). Therefore, it is extremely vital that we consider their functional role in a dynamic and spatiotemporal manner. In this mini-review, we will provide insights on the trafficking patterns of monocytes and how their behavior in distinct tissue compartments governs their function in immune responses (Figure 1).

## Traveling Back in Time: Recognition of the Fetal Monocyte

When van Furth and Cohen's proposal of ontogeny of tissue macrophages arising solely from monocytes (3) was challenged in the early twenty-first century, scientists postulated that adult tissue macrophages were derived from embryonic precursors before birth instead (6, 7, 18). In mice, these embryonic precursors emerged before the development of hematopoietic stem cell (HSC) progenitors and comprised of erythro-myeloid precursors (EMPs) that appear in the yolk-sac blood islands of the embryo at around E7.0 of gestation (19, 20). Importantly, these EMPs could bypass the monocyte stage and give rise directly to primitive macrophages that would seed the organs of the growing embryo (6, 21, 22). However, it was later discovered that upon establishment of the blood circulation, these EMPs migrate and seed the fetal liver at E9.5 of gestation (19, 23, 24), giving rise to multiple myeloid lineage cells, including a very important cell type—the fetal monocyte (25–27).

In mice, fetal monocytes were first reported by Naito et al. and were shown to emerge in the fetal liver around E12.5 before being released into the blood from E13.5 onwards (27, 28). Despite primitive macrophages already occupying the tissue niches at this stage, fetal monocytes were discovered to colonize the remaining open niches of every tissue at E14.5 with the exception of the brain (26, 29–32) (Figure 1A). To date, little is known about the trafficking mechanisms that are adopted by fetal monocytes. Nevertheless, fetal monocyte migration into tissues is independent of the CCR2-CCL2 axis (26) while their egress from the fetal liver is dependent on plasmalemma vesicle-associated protein (PLVAP), which is an endothelium-specific molecule that forms diaphragm-like structures in the fenestrae of the liver sinusoidal endothelium (33) (Figure 1A). Functionally, fetal monocytes share many common traits with adult BM-derived monocytes but have reduced expression of antigen presentation and pathogen recognition-associated genes (26). In contrast to adult monocytes, fetal monocytes also retain a high proliferative capacity in tissues that is CSF-1 receptor independent (29), thereby allowing fetal monocytes to harbor a competitive advantage in replenishing tissue macrophages (34). Further investigations would be required to comprehend how fetal monocytes traffic into tissues and what signals affect their retention in their respective niches as they differentiate into macrophages.

## Monocytes in-Waiting: the Bone Marrow and Spleen

Unlike fetal monocytes that are derived from late EMPs in the fetal liver, adult monocytes originate from HSC progenitors in the BM after birth (7, 35, 36). It was initially thought that Ly6C^hi^ monocytes originated directly from the common monocyte progenitor (cMop) and are poised to leave the BM upon maturing beyond the cMop stage (35). However, contrary to this assumption, recent findings by Chong et al. have demonstrated that cMops undergo an additional step of maturation into a transitional precursor before the ensuing mature monocytes (37). This transitional precursor was termed “transitional pre-monocytes” (TpMos), and was discovered when BM Ly6C^hi^ monocytes were found to contain two distinct subpopulations: (1) the CXCR4^hi^ subpopulation, which constitutes TpMos derived directly from cMops and are immobilized in the BM where they proliferate rapidly to replenish mature monocytes; (2) the CXCR4^lo^ subpopulation, which consists of *bona fide* mature Ly6C^hi^ monocytes that have exited the cell cycle and are readily mobilized from the BM (37) (Figure 1Bi). Since TpMos are highly proliferative and immobilized in the BM under regular circumstances, their presence likely serves as a regulatory checkpoint for the rapid replenishment and prevention of an uncontrolled release of BM monocytes.

In comparison to other myeloid cells (38), monocytes transit quickly through the BM and are released rapidly into the circulation after their last division (39). Their egress and retention in the BM is critically dependent on CCR2-signaling (40, 41) and CXCR4-signaling (37, 42, 43), respectively. Unlike vascular monocytes that are highly motile, Ly6C^hi^ monocytes in the BM parenchyma are comparatively sessile, displaying slow random displacements (44) while being juxtaposed to Nestin^+^ stromal cells (42, 45). Upon sensing of inflammatory stimuli like LPS through Toll-like receptor 4 (45), Nestin^+^ stromal cells express CCL2 (42), which causes BM Ly6C^hi^ monocytes to increase their velocity and displacement (46). This CCL2 exposure also leads to desensitization of monocyte response to CXCL12 (ligand of CXCR4) possibly through internalization of CCR2-CXCR4 complexes, which weakens the CXCR4 anchoring signal and results in their eventual egress (42). Furthermore, only mature Ly6C^hi^ monocytes, and not TpMos, were able to leave the BM under subclinical doses of LPS because TpMos were unable to respond to CCL2 as efficiently as mature Ly6C^hi^ monocytes (37). CX3CR1 was also discovered to regulate Ly6C^hi^ monocyte numbers in the BM after cyclophosphamide-induced myeloablation although their effect is less pronounced than CCR2-signaling (47). While signals governing the release of BM Ly6C^hi^ monocytes are well-documented, mechanisms regulating Ly6C^lo^ monocyte egress are less defined. Nevertheless, it was discovered that Ly6C^lo^ monocytes have very low levels of the CCR2 receptor and thus their egress is more likely to depend on S1PR5 (48).

Besides the BM, monocytes have also been found to reside in the subcapsular red pulp of the spleen as a secondary reservoir (49). In contrast to the BM whose main function lies in immune cell generation from HSC progenitors, the spleen functions mainly as a lymphatic organ (50). Therefore, the steady state monocyte reservoir is not generated in the spleen itself, but derived from circulating monocytes that have entered the spleen (49). Exceptions to this rule, however, do occur in the case of extramedullary hematopoiesis, when monocyte progenitors were found to expand in the spleen during inflammation, contributing to the monocyte reservoir *in situ* (51, 52). More importantly, splenic monocytes can increase their motility and exit into the blood during myocardial infarction via Angiotensin II-signaling and this process is independent of CCR2-signaling (49) (Figure 1Bi). Interestingly, Angiotensin II-dependent recruitment of monocytes into the infarct (a localized area of dead tissue resulting from failure of blood supply) is strictly mediated from the spleen and peripheral circulation, but not from the BM (51). Splenic monocytes were also found to be mobilized to the ovaries where they enhance ovulatory processes (53). Notably, the spleen is also a key site for an alternative source of monocytes in cardiovascular diseases (52, 54, 55), tumor progression (56) and lung ischemia (57). These findings hence suggest that the spleen fulfills the urgent demand of monocytes during inflammation by providing an emergency source, which extends time for the BM to generate more monocytes concurrently.

## Monocytes on-the-go: Navigating Through the Circulatory Highways

Upon entering the circulation, monocytes rely heavily on the circulatory system for transportation to peripheral compartments. Ly6C^hi^ monocytes have a half-life of approximately 20–24 h in the peripheral blood before gradually differentiating into Ly6C^lo^ monocytes (half-life of 48 h in mice; 7 days in humans) via *Nr4a1*-signaling (58–61). Unlike classical Ly6C^hi^ monocytes that roll along vessels, CX3CR1^high^ non-classical Ly6C^lo^ monocytes in mice (62) and their human counterparts (CD14^+^CD16^+^ monocytes) (63) patrol vessels by crawling at a speed of 12 μm/min. Their patrolling behavior is partially mediated by Mac-1 and is highly dependent on CX3CR1-signaling and LFA-1/ICAM-1 or ICAM2 interaction with endothelial cells (62, 64, 65). Furthermore, this patrolling activity is critical for micro-scavenging the luminal surface of vessels and maintaining endothelial integrity (64) (Figure 1Bii). Notably, an increase in atherosclerotic endothelial apoptosis (66), amyloid deposition (67) and tumor metastasis (68) was observed when Ly6C^lo^ monocytes were absent in *Nr4a1*^−/−^ mice. Because of their close interaction with vessels, Ly6C^lo^ monocytes orchestrate the recruitment and activation of neutrophils upon sensing a breach in vascular integrity through TLR7-signaling, which subsequently leads to their retention in the capillaries (64, 69).

**Figure 2:**
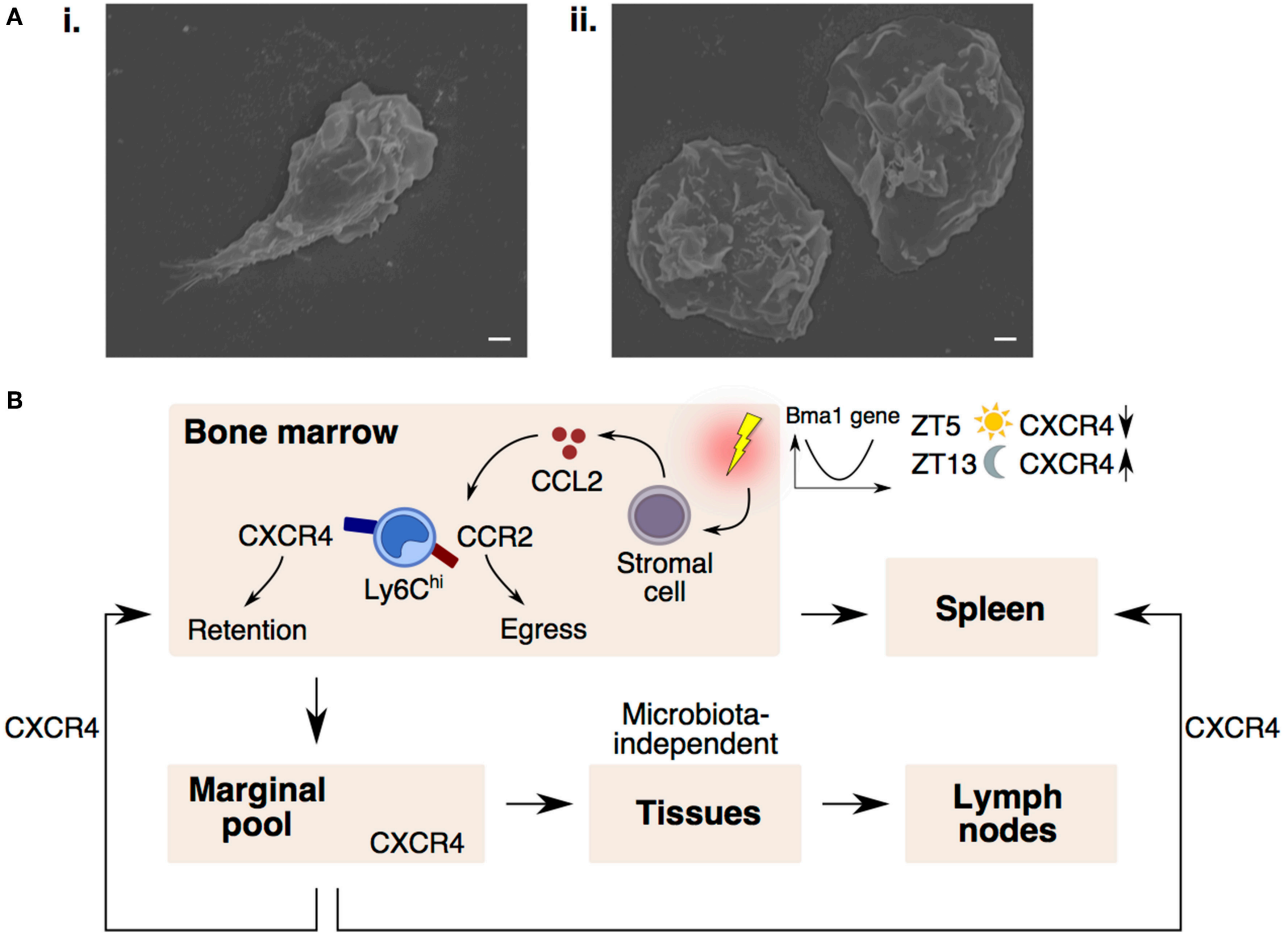
CXCR4 controls monocyte trafficking into different peripheral compartments. **(A)** Scanning electron microscopy images of a Ly6C^hi^ monocyte (i) protruding its pseudopod upon adhering to coverslip and (ii) extending their cytoplasmic membrane when fully adhered to the coverslip. Bars, 1 μm. **(B)** Monocyte egress and retention in the bone marrow is dependent on CCR2-signaling and CXCR4-signaling. Upon sensing inflammatory stimuli, stromal cells release CCL2, desensitizing monocyte response to CXCL12 (CXCR4 ligand), resulting in monocyte entry into the circulation and spleen. In the circulation, CXCR4 regulates steady-state monocyte margination in tissue marginal pools. Monocytes may also extravasate into tissues and lymph nodes in a microbiota-independent manner. CXCR4-signaling also regulates the homing of circulating monocytes back to the bone marrow and spleen. Monocyte numbers display diurnal oscillation that is regulated by the circadian gene, *Bma1*. Lower CXCR4 levels at ZT5 (lights on period) results in more circulating monocytes, whereas higher CXCR4 levels at ZT13 (lights off period) results in higher monocyte retention in the bone marrow.

In contrast to Ly6C^lo^ monocytes that patrol vessels, it is commonly recognized that Ly6C^hi^ monocytes do not interact closely with the endothelium in the steady-state (70). However, exceptions to this rule do occur in vascular beds of distinct peripheral organs. These vascular beds consist of multiple small-caliber microvessels (<5 μm in diameter), which necessitate larger leukocytes (6–8 μm) to deform and physically interact with the endothelium for their transit (71). This phenomenon results in substantial leukocyte retention and the formation of a “marginal pool.” In particular, the lungs represent a major site of leukocyte margination, and classical Ly6C^hi^ monocytes were discovered to form close interactions with the lung vasculature under resting state (37, 72, 73). Ly6C^hi^ monocytes are highly adherent upon contact with surfaces and can be seen to extend their pseudopods upon movement (Figure 2A). Notably, we discovered that CXCR4 regulates steady-state monocyte margination in the lung (37) (Figure 1Bii). Upon endotoxin sensing, classical Ly6C^hi^ monocytes increased their lung transit time (74) by adhering to the endothelium, resulting in increased predisposition towards lung injury that can be reversed with CXCR4 inhibition (37). Apart from the lung, intravital imaging of monocytes in vascular beds of the kidney (75, 76) and liver (77) revealed increased retention of monocytes in the microvasculature during inflammation. Increased adhesion of Ly6C^hi^ monocytes, but not neutrophils, in the brain microvasculature during cerebral malaria is also associated with progressive worsening of clinical symptoms (78). Additionally, the BM was discovered to contain a CX3CR1-dependent marginal pool of monocytes that can be rapidly deployed to the peritoneum (79).

Since the BM is constantly releasing monocytes into the circulation, it is conceivable that a counterbalancing mechanism exists to ensure that circulating monocyte numbers return to homeostasis. Indeed, CXCR4-signaling keeps this homeostasis in check by influencing the spatiotemporal localization of monocytes between the circulation and peripheral compartments (Figure 2B). Notably, circulating monocytes were found to return at a constant rate to the BM and spleen parenchyma in a CXCR4-dependent manner (37). More importantly, the number of circulating monocytes compared to the numbers in the peripheral compartments were found to vary according to circadian rhythmic oscillations, with more monocytes present in the circulation at Zeitgeber 5 (ZT5) than ZT13 in mice (where ZT0 refers to lights on and ZT12 to lights off) (37, 80). This diurnal oscillation of monocyte numbers is regulated by the circadian gene, *Bma1* (80), and also corresponds with diurnal fluctuations in CXCR4 levels on mature monocytes (37), such that absence of CXCR4 also abolishes the diurnal oscillation in monocyte numbers.

## Monocytes Exiting the Highways: Exploring Tissues

The entry of monocytes into tissues is critical for pathogen clearance and wound healing. Furthermore, it is typically acknowledged that their time of entry dictates their function, as ingress of monocytes in the early phase of inflammation is associated with a pro-inflammatory phenotype, while their presence in the later phase corresponds to an anti-inflammatory function (81, 82) (Figure 1Biii). Mediators that attract circulating monocytes into tissues include chemokines, complement components, and products of tissue matrix degradation (83). Since patrolling Ly6C^lo^ monocytes interact closer with the endothelium compared to Ly6C^hi^ monocytes, it is conceived that their migratory dynamics into tissues are quicker than Ly6C^hi^ monocytes. Indeed, Ly6C^lo^ monocytes infiltrate within an hour into inflamed tissues induced by aseptic wounding, irritants or *Listeria monocytogenes* to provide the initial sources of TNF-α and IL-1 (62). In contrast, Ly6C^hi^ monocyte recruitment into tissues typically occurs 24–48 h after injury (84). Their entry into tissues involves vascular rolling, adhesion, and transendothelial migration that has been well-documented (14, 83, 85). Nevertheless, a proportion of Ly6C^hi^ monocytes have also been shown to utilize microhemorrhages to exit blood vessels and enter inflammatory sites rapidly (86). This allows Ly6C^hi^ monocytes to enter the injury site as quickly as neutrophils, where they were found to scout the wound bed randomly before progressively slowing down over a study period of 2.5 h (86). While it is unclear what causes this behavioral change, it is likely that this may be associated with the conversion of Ly6C^hi^ into Ly6C^lo^ monocytes that is critical for wound healing. Indeed, Ly6C^hi^ monocytes entered the injury site and formed a ring-like structure around the injured foci that persisted for 48 h in a model of sterile hepatic injury (77). These Ly6C^hi^ monocytes subsequently differentiated into Ly6C^lo^ monocytes after sensing IL-4 and IL-10 within the ring-like structure. Notably, this phenotypic conversion was critical for monocytes to move further into the injury area and to initiate optimal repair. These findings further highlight the plasticity of monocytes in their functional reprogramming by switching from an inflammatory phenotype to a profile that facilitates wound repair.

Upon entering tissues, infiltrating monocytes progressively alter their phenotype by adopting macrophage characteristics while losing monocyte features, and this gradual differentiation process is known as the classical “monocyte waterfall” effect (8, 87, 88). Besides replacing certain residential macrophages in the steady-state (6, 18), monocytes may also differentiate into TNF/iNOS-producing DCs (Tip-DCs) (89), wound-associated macrophages (WAMs) (90) or tumor-induced myeloid suppressor cells (91). However, *bona fide* classical monocytes have also been found to remain undifferentiated in the tissue at resting state (92). These monocytes extravasated constitutively into tissues and lymph nodes in a CCR2-dependent manner and retained most of their existing monocyte transcriptional profile. Nevertheless, these Ly6C^hi^ monocytes increased their expression of MHCII, co-stimulatory molecules and CCR7, suggesting that these cells survey the tissue environment for antigens to transport to draining lymph nodes in the steady state. Since monocyte extravasation into tissues in the steady-state was found to be microbiota-independent (92), it would be interesting to determine the specific mechanisms that dictate their migration into tissues and the factors that preserve their profile in these circumstances.

## Conclusion and Future Perspectives

Despite being described in many important studies in the last century, our comprehension of monocyte biology has only taken a substantial leap in the past decade upon the advent of highly sophisticated imaging techniques that complement the current use of biochemistry, cell biology and genetic tools. More importantly, 2P-IVM has unveiled critical trafficking mechanisms that may have important implications for future vaccine designs/therapeutic strategies. In particular, the specific kinetics of monocyte trafficking in different tissue compartments and their interaction with other immune cells will allow scientists to optimize their drug administration and design according to these dynamics. For example, clinicians who aim to reduce tissue inflammation may take advantage of the knowledge that non-classical monocytes recruit neutrophils in the early stages of inflammation (64). Therefore, selecting specific drugs that target molecules only on non-classical monocytes, instead of both monocyte subsets, may help to reduce the likelihood of any off-target effects and secondary infections during long periods of therapy. While 2P-IVM has provided valuable insight, major technical bottlenecks still exist against gaining a global understanding of these cells in chronic disease states. These issues are due to the highly plastic nature of monocytes, which may include the loss of fluorescence signal as they differentiate into monocyte-derived cells. Furthermore, their differentiated phenotypes are distinct in various chronic disease settings (13, 93). In this regard, a combination of tools that would enable researchers to identify monocyte-derived cells with greater spatiotemporal specificity would be beneficial in addressing these issues. In particular, multiplex immunofluorescence techniques (94, 95) in a histo-cytometry setting (96, 97) that involves optically cleared large tissue samples (98) would provide a global view of their localization and interaction with other immune cells. Furthermore, refining image analysis methods that deal with large volumes of data, such as using a hue-saturation-brightness-based surface creation to streamline multi-channel image cytometry for three-dimensional images (99), would allow us to uncover new markers on monocyte-derived cells that can be used to generate improved fluorescent-tagged mice. Importantly, while transcriptomic studies have shown mouse and human monocytes to be homologous, a reverse pattern in certain genes such as TREM-1, CD36, CXCR4, and CD9 was also discovered (12). Therefore, future work adopting humanized mice for 2P-IVM studies, is warranted to verify if trafficking mechanisms of mouse monocytes are similar to that in humans. Taken together, we believe that the combination of these state-of-the-art imaging tools in future studies will provide further insight into the temporal and spatial landscape of monocytes that could hold the key for future biomarker and therapeutic discoveries.

## Author Contributions

YCT, JLD, LGN and SZC wrote and conceptualized the manuscript. YCT did the figures.

### Conflict of Interest Statement

The authors declare that the research was conducted in the absence of any commercial or financial relationships that could be construed as a potential conflict of interest.

## Abbreviations

BM: bone marrow
cMop: common monocyte progenitor
CSF-1: colony stimulating factor-1
DC: dendritic cell
EMP: erythro-myeloid precursor
HSC: hematopoietic stem cell
IL: interleukin
iNOS: inducible nitric oxide synthase
LPS: lipopolysaccharide
MHC: major histocompatibility complex
PLVAP: plasmalemma vesicle-associated protein
S1PR5: sphingosine-1-phosphate receptor 5
2P-IVM: two-photon intravital imaging
TNF: tumor necrosis factor
TpMo: transitional pre-monocyte
ZT: Zeitgeber.
